# Rational Design of an *In‐Situ* Polymer‐Inorganic Hybrid Solid Electrolyte Interphase for Realising Stable Zn Metal Anode under Harsh Conditions

**DOI:** 10.1002/anie.202401987

**Published:** 2024-04-18

**Authors:** Ruwei Chen, Wei Zhang, Chaohong Guan, Yundong Zhou, Ian Gilmore, Hao Tang, Zhenyu Zhang, Haobo Dong, Yuhang Dai, Zijuan Du, Xuan Gao, Wei Zong, Yewei Xu, Peie Jiang, Jiyang Liu, Fangjia Zhao, Jianwei Li, Xiaohui Wang, Guanjie He

**Affiliations:** ^1^ Department of Chemistry University College London London WC1E 7JE UK; ^2^ State Key Laboratory of Pulp and Paper Engineering South China University of Technology Guangzhou 510641 China; ^3^ National Physical Laboratory Hampton Road Teddington TW11 0LW UK; ^4^ University of Michigan-Shanghai Jiao Tong University Joint Institute Shanghai Jiao Tong University Shanghai 200240 China; ^5^ Electrochemical Innovation Lab, Department of Chemical Engineering University College London London WC1E 7JE UK

**Keywords:** Aqueous Zinc-Ion batteries, Zinc Anode, Solid Electrolyte Interphase, Polymer-Inorganic SEI

## Abstract

The in‐depth understanding of the composition‐property‐performance relationship of solid electrolyte interphase (SEI) is the basis of developing a reliable SEI to stablize the Zn anode‐electrolyte interface, but it remains unclear in rechargeable aqueous zinc ion batteries. Herein, a well‐designed electrolyte based on 2 M Zn(CF_3_SO_3_)_2_‐0.2 M acrylamide‐0.2 M ZnSO_4_ is proposed. A robust polymer (polyacrylamide)‐inorganic (Zn_4_SO_4_(OH)_6_.xH_2_O) hybrid SEI is in situ constructed on Zn anodes through controllable polymerization of acrylamide and coprecipitation of SO_4_^2−^ with Zn^2+^ and OH^−^. For the first time, the underlying SEI composition‐property‐performance relationship is systematically investigated and correlated. The results showed that the polymer‐inorganic hybrid SEI, which integrates the high modulus of the inorganic component with the high toughness of the polymer ingredient, can realize high reversibility and long‐term interfacial stability, even under ultrahigh areal current density and capacity (30 mA cm^−2^~30 mAh cm^−2^). The resultant Zn||NH_4_V_4_O_10_ cell also exhibits excellent cycling stability. This work will provide a guidance for the rational design of SEI layers in rechargeable aqueous zinc ion batteries.

## Graphical abstract

A robust polymer‐inorganic SEI is in situ constructed on Zn anode through controllable polymerization and coprecipitation mechanism. Compared with bare Zn and recent in situ SEIs, polymer‐inorganic SEI combines both superiorities of polymer phases for high toughness and inorganic phases for high modulus, enabling stable electrode/electrolyte interface even under harsh conditions.

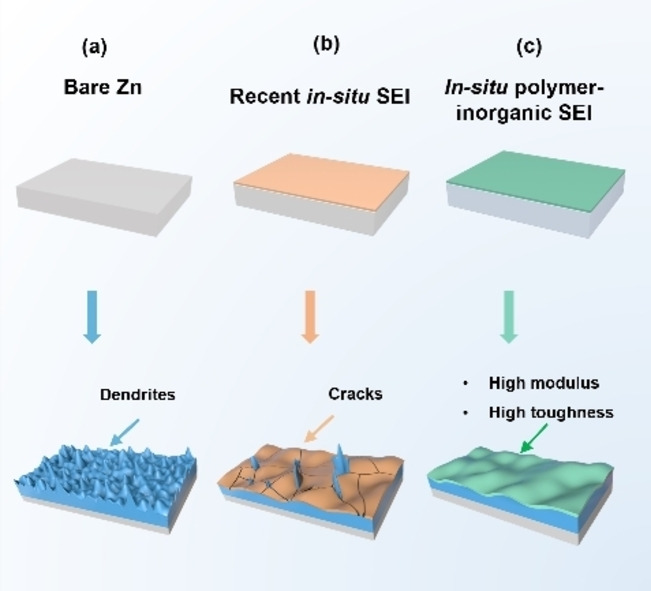

## Introduction

**Scheme 1:**
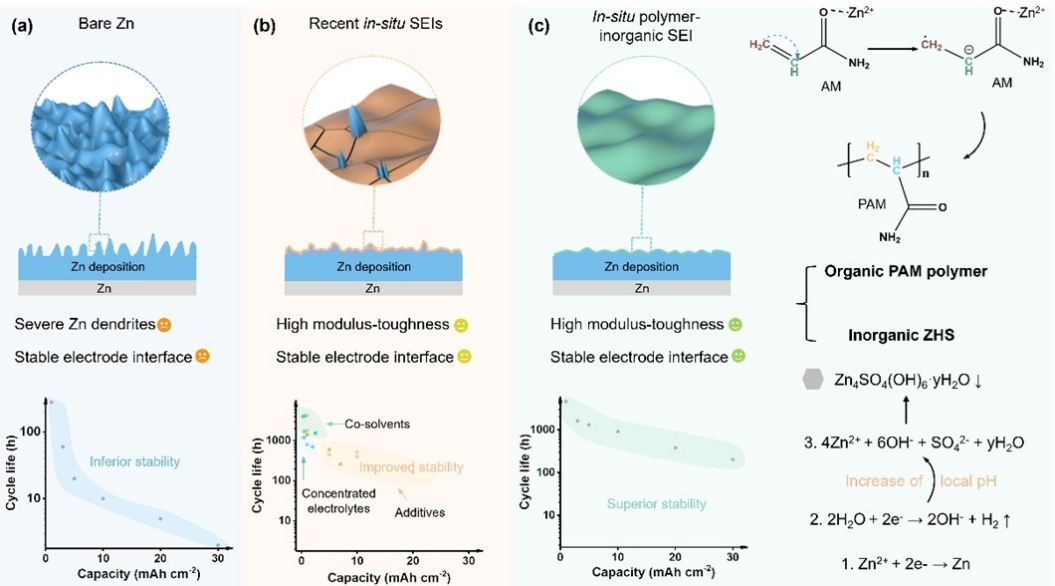
Schematic illustrations of (a) Zn deposition without protection; (b) Zn deposition with recent SEIs; (c) Zn deposition with our in situ formed polymer‐inorganic SEI, and in situ formation mechanism.

Rechargeable aqueous zinc‐ion batteries (RAZBs) have been regarded as promising candidates for large‐scale energy storage applications because of their low cost, high safety, and high theoretical capacity (820 mAh g^−1^ and 5855 mAh cm^−3^) in terms of mildly acidic aqueous electrolytes and Zn metal anodes.[^1^, ^2^, ^3^] However, interfacial stability and compatibility in RAZBs are still needed to be optimized under operation due to the electrochemical activity of the Zn metal anode and its spontaneous side reactions in aqueous electrolytes.[^4^, ^5^] Correspondingly, notorious hydrogen evolution reaction (HER), Zn corrosion, and dendrite growth continue to occur at the Zn anode‐electrolyte interface, which seriously destroy the reversibility and lifespan of RAZBs (Scheme 1a).

Building a protective SEI is a widely adopted approach to stabilize the Zn anode‐electrolyte interface, as it can block the direct contact between the Zn anode and electrolyte.[^6^, ^7^, ^8^] Nevertheless, most of these artificial SEIs relying on complex or ex situ physical coatings generally suffer from cracking or detaching from the Zn surface during repeated cycling due to the volume variation of Zn.[^9^, ^10^] When encountered with a similar dilemma of the Li‐metal batteries, the in situ formed SEI on the Li anode derived from the reductive decomposition of anions and organic solvents could not only seamlessly ensure cohesion of electrode but also the self‐healable ability prevent the crack situation and ensure the stable anode‐electrolyte interface.[^11^, ^12^] Unfortunately, considering much higher redox potentials of HER (0 V vs. standard hydrogen electrode (SHE)) and Zn^2+^/Zn (−0.76 V vs. SHE) compared with that of Li^+^/Li (−3.04 V vs. SHE), routine anions and solvents are difficult to decompose reductively before Zn deposition and HER.^[13]^ Hence, it is highly desired but very challenging to in situ build a reliable SEI on the Zn anode in aqueous electrolytes.

Constrained by this inherent limitation, only a few successful attempts have been achieved to in situ build SEIs on the Zn anode through concentrated electrolytes, electrolyte additives, and organic co‐solvents. For example, Alshareef's group proposed an concentrated electrolyte of 0.5 m Zn(ClO_4_)_2_ with 18 m NaClO_4_. A Cl‐containing SEI was in situ formed on the Zn surface to enhance the Zn reversibility.^[14]^ Guo's group demonstrated the in situ formation of a hopeite‐SEI (Zn_3_(PO_4_)_2_.4H_2_O) on the Zn surface by a Zn(H_2_PO_4_) additive in 1 M Zn(CF_3_SO_3_)_2_.^[15]^ Very recently, Yang et al. reported a novel hydrous organic electrolyte consists of hydrated Zn(BF_4_)_2_ and ethylene glycol, which leads to the in situ formation of a fluoride‐SEI on the Zn surface.^[16]^ Benefiting from these in situ SEIs, enhanced interfacial stability and improved Zn reversibility have been achieved.[^17^, ^18^, ^19^] Unfortunately, these developed SEIs are still prone to break at high current densities and areal capacities with huge Zn volume variations (Scheme 1b), resulting in gradual loss of protective function.[^20^, ^21^]

Organic‐inorganic composites, especially polymer‐inorganic hybrid materials, combine both superiorities of polymer phases for flexibility and inorganic phases for strength, which might be more prospective as SEIs for RAZBs when facing huge volume variation at large areal capacities.[^22^, ^23^] However, such an effective in situ formed polymer‐inorganic hybrid SEI for the Zn anode is never reported due to the lack of feasible implementation routes restrained by abovementioned higher redox potentials of HER and Zn deposition.[^24^, ^25^] Importantly, except for lacking of feasible implementation routes, the formation mechanisms and the specific compositions of currently reported SEIs are unclear owing to the uncontrollable decomposition reactions of anions or additives, let alone the underlying SEI composition‐property‐performance relationship.[^26^, ^27^, ^28^] The clarification of these scientific issues is critical for understanding, developing, and applying of SEIs in RAZBs. Therefore, in situ constructing a mechanically robust polymer‐inorganic hybrid SEI with clear formation mechanism and compositions and in‐depth understanding the SEI composition‐property‐performance relationship are highly desired.

To achieve this ambitious goal, for the first time, a facile and scalable strategy was proposed to in situ build a hybrid polymer inorganic SEI on the Zn anode by introducing acrylamide (AM) and ZnSO_4_ additives into a common aqueous electrolyte (2 M Zn(CF_3_SO_3_)_2_). Catalyzed by Zn species, unsaturated AM molecules in situ polymerize into polyacrylamide macromolecules (PAM) to form highly flexible polymer species on the Zn anode. The introduction of ZnSO_4_ facilitates the in situ formation of inorganic Zn_4_SO_4_(OH)_6_.xH_2_O (ZHS) component through a self‐terminated coprecipitation of SO_4_^2−^ with Zn^2+^ and OH^−^ originating from HER and the increase of local pH during the initial plating process (Scheme 1c). Consequently, a hybrid polymer‐inorganic SEI consists of PAM macromolecules and inorganic ZHS was successfully in situ constructed on the Zn anode through the polymerization and coprecipitation reactions (Figure S1), which is totally different from other methods designing in situ SEIs via uncontrollable decomposition of anions or additives. To understand the function mechanism, the SEI compositions, SEI properties, and electrochemical performances were systematically investigated and correlated. The results show that the polymer‐inorganic SEI integrating high modulus of the inorganic component with high toughness of the organic polymer ingredient can realize long‐term interfacial stability. Therefore, the Zn||Cu asymmetric cell with polymer‐inorganic SEI displays a high average Coulombic efficiency of 99 %, indicating highly reversible Zn plating/stripping behavior. The Zn||Zn symmetric cell delivers a stable cycling for over 4600 h at 1 mA cm^−2^~1 mAh cm^−2^. Stable long‐term cycling is still maintained even under harsh plating/stripping conditions (30 mA cm^−2^~30 mAh cm^−2^), outperforms most of other reported works. Besides, improved electrochemical performances can also be achieved in Zn||NH_4_V_4_O_10_ full cells by taking the advantages of the hybrid SEI.

## Results and Discussion

**Figure 1:**
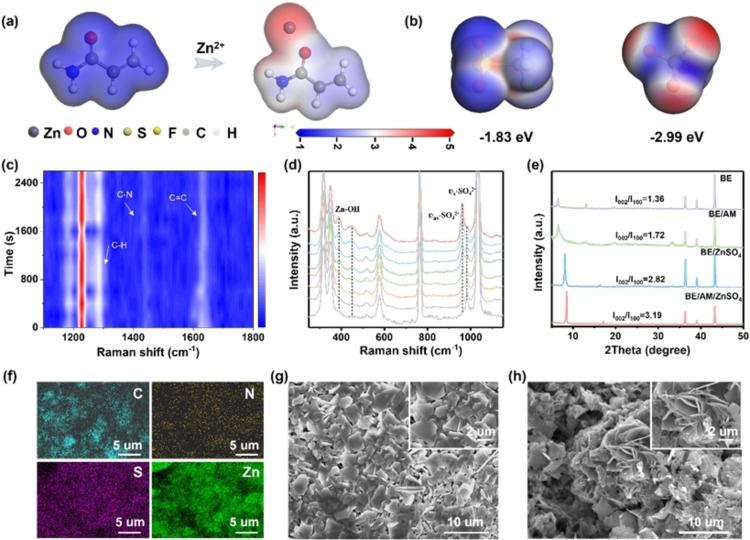
Formation mechanism of the polymer‐inorganic SEI. (a) Molecular electrostatic potential maps of AM and AM binding to Zn^2+^. **b** Binding energies of Zn^2+^ between SO_4_^2−^ and CF_3_SO_3_^−^. (c–d) In situ Raman spectra of the Zn anode surface at different plating time. (e) XRD patterns of Zn anodes after cycling in different electrolytes. (f) EDX mapping images of cycled Zn anodes with polymer‐inorganic SEI. (g) SEM images of cycled Zn anodes with polymer‐inorganic SEI. (h) SEM images of cycled bare Zn anodes.

The formation mechanism of the polymer‐inorganic SEI was investigated by theoretical calculations and experimental studies. AM molecules possess a high binding energy on Zn species.^[29]^ The adsorbed Zn species can withdraw electrons from the carbonyl group along with enhanced conjugated‐electron delocalization, making the unsaturated C=C form a free radical intermediate which subsequently initializes the chain propagation under electrochemical reduction (Figure 1a). Reaction free energies were evaluated for this polymerization process, where a lower free energy was demonstrated during the reaction with Zn^2+^, indicating its thermodynamically favored polymerization process in the presence of Zn^2+^ catalyst (Figure S2).^[30]^ From the in situ Raman spectra, the characteristic peaks of AM are observed at 1286 cm^−1^ (C−H bending), 1440 cm^−1^ (C−N stretching), and 1625 cm^−1^ (C=C stretching), respectively (Figure 1c).^[31]^ Importantly, the peak assigned to C=C stretching gradually weakens and even disappears when the plating time is prolonged. These results further prove that AM monomer gradually polymerizes to PAM, resulting in the formation of polymer layer on the Zn surface.

Theoretically, Zn metal is thermodynamically unstable in 2 M Zn(CF_3_SO_3_)_2_ electrolyte (BE). Inevitable HER accompanied by the increased local pH value leads to the formation of Zn_4_(CF_3_SO_3_)_2_(OH)_6_.xH_2_O (Figure 1e). However, the as‐formed Zn_4_(CF_3_SO_3_)_2_(OH)_6_.xH_2_O is randomly distributed on the Zn surface and cannot form an inorganic SEI to stabilize electrolyte‐anode interface.^[32]^ To accelerate the formation of an inorganic SEI, ZnSO_4_ additive was introduced into BE.^[32]^ According to density functional theory (DFT) calculations, SO_4_^2−^ has higher binding energy with Zn^2+^ due to its higher electronegativity, which enables the preferential formation of ZHS rather than the Zn_4_(CF_3_SO_3_)_2_(OH)_6_.xH_2_O when the critical solubility OH^−^ is reached (Figure 1b). In addition, the saturated concentration in BE is around 0.2 M, and further increasing the content of ZnSO_4_ will lead to precipitation. Therefore, the nearly saturated ZnSO_4_ and the low solubility (k_sp_=2.51×10^−56^) will stimulate the generation of ZHS, which is conducive to the formation of a uniform ZHS‐based SEI.^[17]^ According to the in situ Raman spectra, a peak located at 984 cm^−1^ assigned to the symmetric stretching vibration of dissociated SO4^2−^, where this peak gradually weakens when the plating time is prolonged (Figure 1d).^[33]^ Meanwhile, new peaks located at 389, 451 and 960 cm^−1^ corresponding to the Zn−OH coordination and the fundamental vibration frequency of SO_4_^2−^ with reduced symmetry appear and gradually strengthen along with increased plating time (Figure 1d). These new peaks are fingerprints of ZHS, confirming the generation of ZHS.^[34]^

X‐ray diffraction (XRD) measurement was then conducted to further study the formation mechanism. As shown in Figure 1e, the peaks at 6.5° from Zn_4_(CF_3_SO_3_)_2_(OH)_6_.xH_2_O were observed in BE and BE/AM without ZnSO_4_ additives, indicating the occurrence of side reactions.^[35]^ In contrast, obvious peaks located at 8.4° assigned to ZHS rather than Zn_4_(CF_3_SO_3_)_2_(OH)_6_.xH_2_O were observed in BE/ZnSO_4_ and BE/AM/ZnSO_4_ (DE), further confirming the preferential generation of ZHS, which facilitates the formation of uniform ZHS‐based SEI and thereby inhibits side reactions including Zn_4_(CF_3_SO_3_)_2_(OH)_6_.xH_2_O generation.^[36]^ As a consequence, a well‐designed polymer‐inorganic SEI consisting of uniform ZHS flakes connected by PAM was successfully constructed on the Zn anode surface (Figure 1g). Moreover, EDX mapping images of C, N, S, and Zn elements further prove the successful formation of the polymer‐inorganic SEI and the uniform distribution of the polymer and inorganic ingredients (Figure 1f). By comparison, the Zn anode after cycling in BE shows a rough surface with obvious Zn dendrites (Figure 1h).

**Figure 2:**
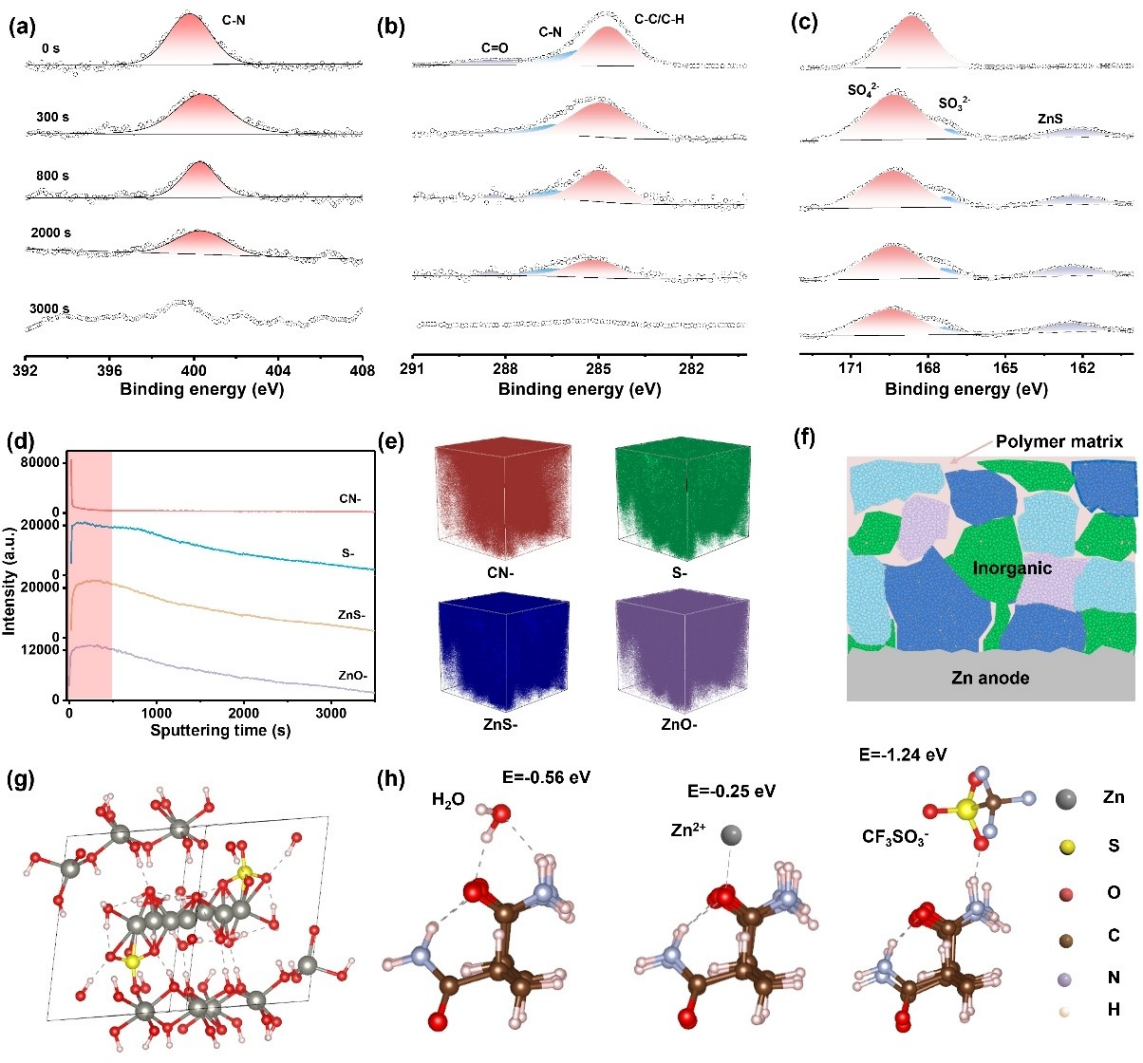
Composition characterization of the polymer‐inorganic SEI. X‐ray photoelectron spectroscopy (XPS) of (a**)** N 1s, (b) C 1s, and (c) S 2p with different durations of Ar^+^ sputtering. The time‐of‐flight secondary‐ion mass spectrometry (TOF‐SIMS) analysis. (d) Intensity profiles of CN^−^, S^−^, ZnS^−^, and ZnO^−^. The red shaded area is to highlight obvious changes. (e) Three‐dimensional (3D) render images of CN^−^, S^−^, ZnS^−^, and ZnO^−^ species. The images were not tomographically corrected and so the surface appears flat.^[37]^ (f) A schematic diagram of the structure model of the polymer‐inorganic SEI. (Different colours represent the same inorganic component) (g) DFT calculation of the typical Zn^2+^‐diffusion path in inorganic ZHS. (h) DFT calculation of the interactions of electrolyte components on PAM polymer.

After clarifying the formation mechanism, the structures and compositions of the in situ formed polymer‐inorganic SEI were further investigated by XPS and TOF‐SIMS analysis with various sputtering times. The N 1s and C 1s spectra in Figure 2a–2b revealed that an organic layer, containing C−N, C−C/C−H, and C=O, was in situ formed on the Zn anode surface. This composition information is consistent with the structure of PAM. In addition, the intensity of N 1s and C 1s peaks decreased gradually and even vanished with increasing sputtering time, indicating the gradient distribution of PAM component, which mainly existed on the top surface of SEI (Figure 2a–2b). The S 2p peak indicates the presence of inorganic component, arising from the co‐precipitation of sulfur containing species with Zn^2+^ and OH^−^ (Figure 2c). This inorganic component mainly containing SO_4_^2−^ species was observed throughout the whole sputtering process. The intensity profiles and 3D render images also provide strong support to above conclusions, where the intensities of organic fragment (CN^−^) drops rapidly with increasing sputtering time while the inorganic fragments (S^−^, ZnS^−^, ZnO^−^) dropped slowly (Figure 2d–2e, Figure S3 and Figure S4a). Moreover, the presence of characteristic peaks indexed to SO_4_^2−^, −NH_2_, and −CONH_2_ in FTIR spectra further confirm the composition of polymer‐inorganic SEI (Figure S4b). Based on these characterizations, a structure model of the hybrid SEI was constructed, which consists of a PAM polymer‐inorganic ZHS dominated top layer and an inorganic ZHS dominated inner layer (Figure 2f).

DFT calculations were subsequently employed to evaluate the working mechanism of the polymer‐inorganic SEI layer. Figure 2g shows the possible diffusion channel in the tunnel‐like framework of inorganic ZHS. The corresponding Zn^2+^ diffusion energy barrier for this pathway was calculated to be 0.52 eV, which is relatively low considering the generally high energy barriers of multivalent cation migration, suggesting its fast Zn^2+^ diffusion behavior (Figure S5a).[^15^, ^38^] Figure 2h exhibits the calculated models of the interaction between different electrolyte components and PAM polymer. The binding energy of different electrolyte components on the polymeric structure follows the order of SO_4_^2−^<CF_3_SO_3_^−^<H_2_O<Zn^2+^ (Figure 2h and Figure S5b). The high binding energy of the PAM directly demonstrates that the polymeric structure possesses a strong adsorption capability towards SO_4_^2−^, CF_3_SO_3_^−^, and H_2_O at the electrode‐electrolyte interphase, which is in favor of Zn^2+^ desolvation process.^[39]^ Moreover, Zn symmetric cells with polymer‐inorganic SEI have significantly increased Zn^2+^ transference number (*t*_Zn2+_) and dramatically decreased activation energy, suggesting that the polymer‐inorganic SEI facilitates fast Zn^2+^ desolvation and Zn^2+^ diffusion processes, which further corresponds to the calculation results (Figure S6–S7).^[40]^

**Figure 3:**
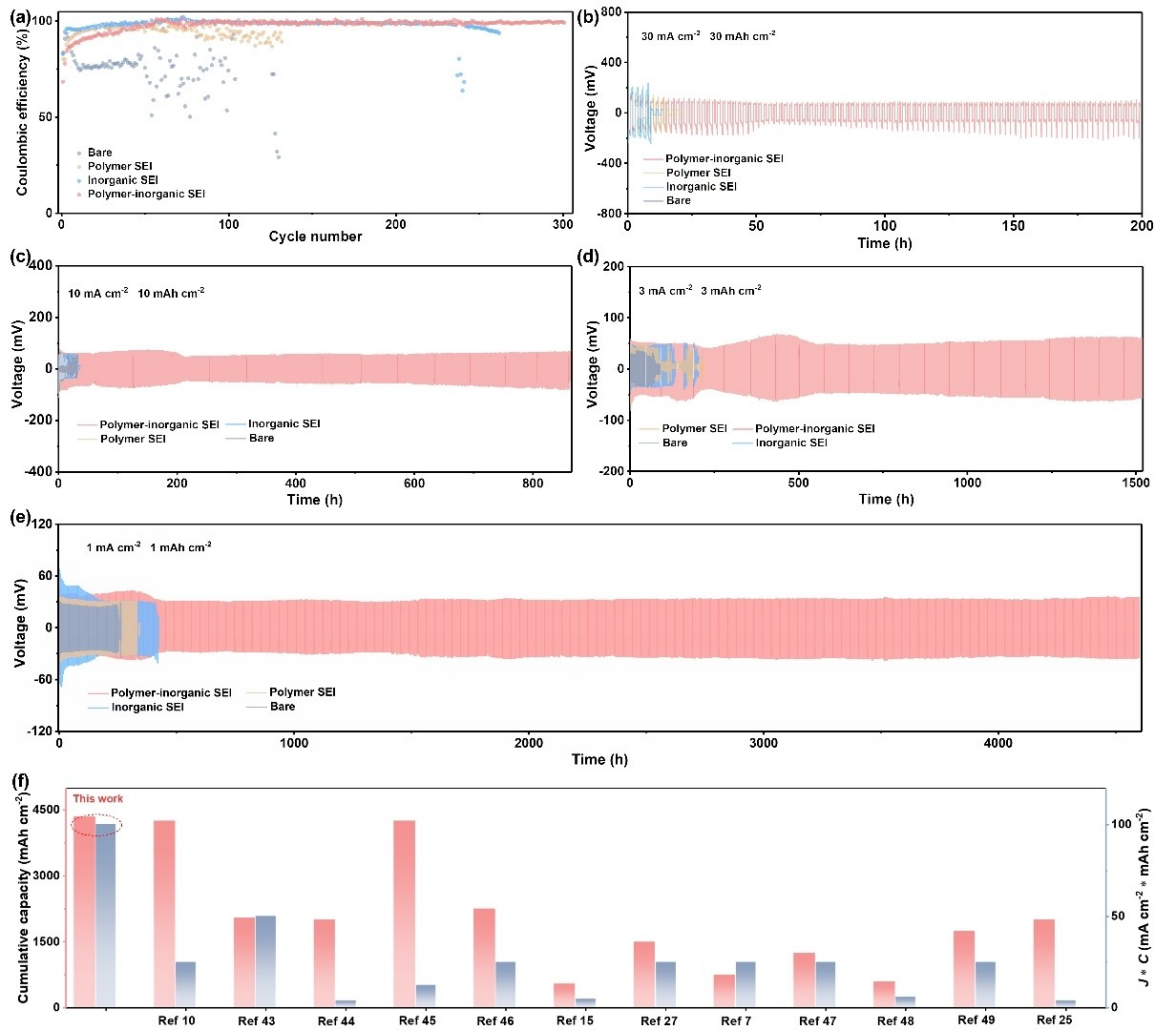
Zn plating/stripping behaviors of Zn anodes with different SEIs. (a) Coulombic efficiency (CE) of Zn||Cu asymmetric cells. Long‐term cycling performance of Zn||Zn symmetric cells at different current densities and areal capacities: (b) 30 mA cm^−2^ ~30 mAh cm^−2^, (c) 10 mA cm^−2^~10 mAh cm^−2^, (d) 3 mA cm^−2^ ~3 mAh cm^−2^, (e) 1 mA cm^−2^~1 mAh cm^−2^. (f) Comparison of cumulative capacity and *J* * *C* of Zn||Zn symmetric cells from state‐of‐the‐art works.

To clarify the effect of the SEIs on Zn plating/stripping behaviors, both Zn||Cu and Zn||Zn cells were assembled. The Zn||Cu cell in BE displays inferior cycling performance with very low average CE and short lifespan, ascribed to severe side reactions at electrolyte‐Zn interface without any SEI protection (Figure 3a). Although the introduction of polymer SEI can stabilize cycling with increased CE, the CE fluctuates obviously with prolonged cycling duration, suggesting limited protection of the polymer SEI. The introduction of inorganic SEI substantially improves the CE and cycling lifespan (Figure 3a). However, the CE fluctuates and deteriorates after cycling over 220 cycles, indicating the inorganic SEI also gradually loses its protection function after a long‐term cycling. Strikingly, a high average CE with dramatically enhanced cycling lifespan achieved in polymer‐inorganic SEI, suggesting significantly improved interfacial stability and reversibility.^[41]^

The efficiency of different SEIs toward the electrochemical stability of Zn was further evaluated by long‐term galvanostatic cycling of Zn||Zn symmetric cells. As shown in Figure 3e, the symmetric cell with polymer‐inorganic SEI exhibits an extraordinary cycle life of over 4,600 h at a current density of 1 mA cm^−2^ and an real capacity of 1 mAh cm^−2^. Conversely, the voltage profile of bare Zn presents obvious fluctuations after only 400 h, and short‐circuit occurred subsequently. In addition, the symmetric cells with only polymer SEI or inorganic SEI show slightly improved cycle life, which is consistent with above Zn||Cu results. To meet practical application requirements and further verify the effects of the SEIs, the cycling performance at high current densities and high areal capacities was also evaluated. The symmetric cell with polymer‐inorganic SEI was able to stably cycle over 1500 h at a current density of 3 mA cm^−2^ and an areal capacity of 3 mAh cm^−2^ (Figure 3d). Even under a high current density of 10 mA cm^−2^ and a high areal capacity of 10 mAh cm^−2^, the symmetric cell with polymer‐inorganic SEI could maintain stable plating/stripping behaviors for near 900 h and possess a low overpotential of ~50 mV, while the symmetric cells with bare Zn, polymer SEI or inorganic SEI suffer from short‐circuit at the initial cycling stage (Figure 3c). The initial voltage fluctuations originate from the evolution of a more uniform, stable, and compact polymer‐inorganic SEI (Figure S8–S10). Remarkably, under very harsh conditions of 30 mA cm^−2^ with an ultrahigh areal capacity of 30 mAh cm^−2^, the symmetric cell with polymer‐inorganic SEI still maintains a unique cycling behavior up to 200 h (Figure 3b and Figure S11). It is worth noting that the overpotential difference on both sides of the symmetric cell is caused by the difference in the initial stripping/plating behavior.^[42]^ This superior stability achieved in our work outperformed most of the reported Zn metal anodes working at high current densities and areal capacities, demonstrating the critical role of polymer‐inorganic SEI to stabilize the electrolyte‐Zn anode interface (Figure 3f and Table S1).[^7^, ^10^, ^15^, ^25^, ^27^, ^43^, ^44^, ^45^, ^46^, ^47^, ^48^, ^49^]

**Figure 4:**
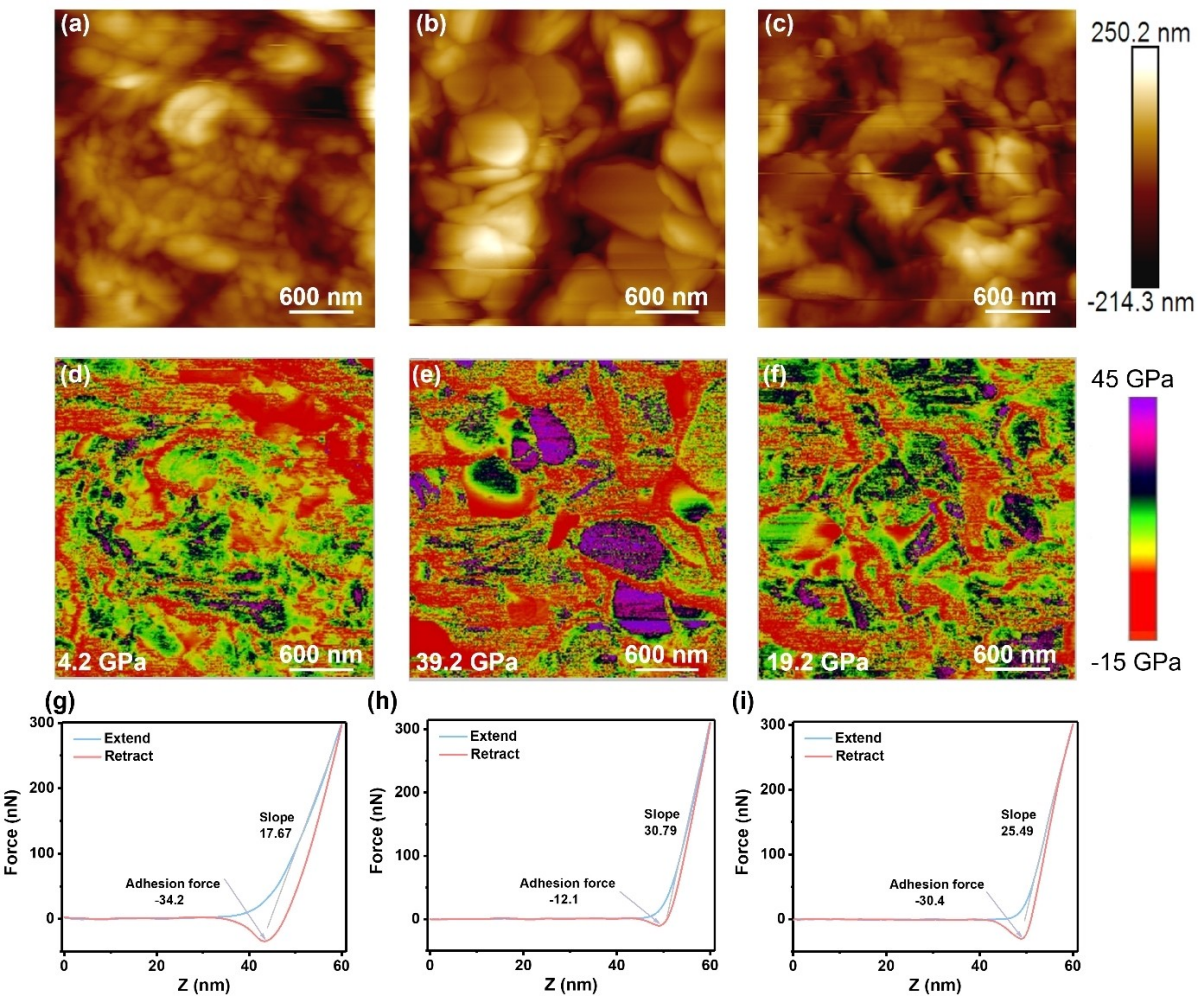
Characterizations of mechanical properties of different SEIs by atomic force microscopy (AFM). AFM topography image of (a) polymer SEI, (b) inorganic SEI, and (c) polymer‐inorganic SEI. DMT modulus and corresponding average Young's modulus of (d) polymer SEI, (e) inorganic SEI, and (f) polymer‐inorganic SEI. Force‐distance curves of (g) polymer SEI, (h) inorganic SEI, and (i) polymer‐inorganic SEI.

To further understand the underlying mechanism behind electrochemical performance, SEIs composition‐mechanical properties‐electrochemical performance were correlated. AFM technique is becoming a crucial platform for the study of lithium‐ion batteries, enabling the versatile characterization of the morphological, mechanical, local‐electrochemical properties of battery materials.^[50]^ In its most common form, AFM provides topographical information of a sample by measuring the displacement of a laser reflected from the back of cantilever attached to the probe. Figure 4a shows the topography image ofthe Zn anode with polymer SEI, it gives a relatively flat surface with granular features. The surface of Zn anodes was covered by irregular flakes after cycling in inorganic SEI forming electrolyte (Figure 4b). In the case of the polymer‐inorganic SEI, the morphology combines the features of polymer and inorganic SEIs (Figure 4c).

In addition, AFM also provides the opportunity to evaluate the mechanical properties of a sample, but it is rarely performed in aqueous zinc‐ion batteries. The deflection of the cantilever as it approaches, interacts with and retracts from the sample gives information about the interaction between the tip and the sample.[^51^, ^52^] In this form, both attractive and repulsive forces are manifestation of mechanical properties. Figure 4g shows the force curves of polymer SEI as a function of Z height during the approaching and retracting cycle. The repulsive force increases slowly when the tip approaches the sample, giving the smallest slope of 17.67. The measured largest attractive force of −34.2 nN is adhesion force when the tip retracts from the sample. The smallest slope and largest adhesion force identify the polymer SEI is soft and flexible along with a low Young's modulus (4.2 GPa, Figure 4d). Such low‐strength polymer SEI can be easily penetrated by Zn dendrites and thereby lose the protective function, which explains the CE of the asymmetric cell with polymer SEI fluctuates obviously with prolonged cycling duration (Figure 3). It should be noted that all force curves and Young's moduli are the average values obtained from six spots (Figure S12). As shown in Figure 4h, the inorganic SEI exhibits the largest slope of 30.79 and smallest adhesion force of −12.1 nN, and the retracting curve nearly overlaps with the approaching curve, suggesting the inorganic SEI is rigid with the highest Young's modulus (39.2 GPa, Figure 4e). Although this high‐strength inorganic SEI is capable of suppressing Zn dendrites growth, the brittle characteristic makes it prone to breakage especially at high current density and areal capacity with huge volume variations. Therefore, the Zn||Cu and Zn||Zn cells also show unsatisfactory reversibility and stability (Figure 3). In contrast, the polymer‐inorganic SEI simultaneously integrates a large slope (25.49) with a large adhesion force (−30.4 nN), possessing a decent Young's modulus (19.2 GPa) (Figure 4f and Figure 4i). This feature points that the well‐designed polymer‐inorganic SEI can combine the high modulus of the inorganic component with high toughness of the polymer ingredient, enabling the capacity of suppressing dendrites growth while accommodating the volumetric change. As a consequence, the Zn anode with this polymer‐inorganic SEI is able to realize high reversibility and superior long‐term stability even at harsh conditions.

**Figure 5:**
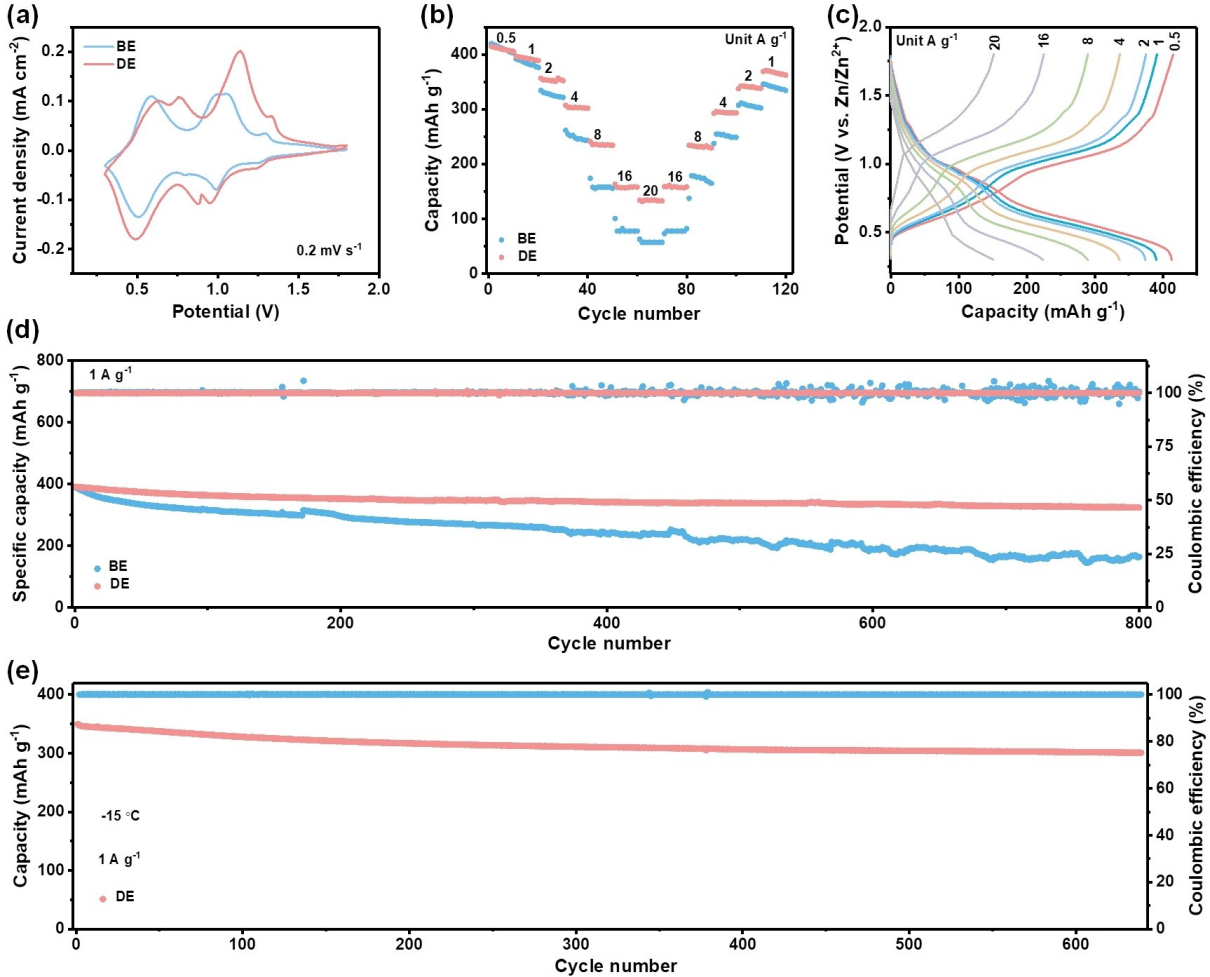
Electrochemical performance of Zn||NH_4_V_4_O_10_ batteries. (a) CV curves of Zn||NH_4_V_4_O_10_ batteries in DE and BE at 0.2 mV s^−1^. (b) Comparison of rate performances in different electrolytes. (c) Corresponding voltage profiles in DE. (d) Comparison of the cycling performance at 1 A g^−1^. (e) Cycling performance of Zn||NH_4_V_4_O_10_ batteries in DE at low temperature.

To investigate the feasibility of DE for practical application, Zn||NH_4_V_4_O_10_ full cells were assembled. The NH_4_V_4_O_10_ cathodes display multiple redox couples in both electrolytes, which are attributed to the Zn^2+^/H^+^ co‐insertion mechanism (Figure 5a). In comparison, the NH_4_V_4_O_10_ cathode presents more steady CV profiles in DE (Figure S13). As shown in Figure 5b, the rate performances were evaluated at various current densities. The Zn||NH_4_V_4_O_10_ full cell in DE exhibits an excellent rate performance with capacities of 415.5, 397.4, 354.1, 304.5, 236.4, and 158.3 mAh g^−1^ at 0.5, 1, 2, 4, 8, 16 A g^−1^, respectively (Figure 5c). Even at a large current density of 20 A g^−1^, a capacity of 135.9 mAh g^−1^ can still be retained. In contrast, the Zn||NH_4_V_4_O_10_ full cell only shows a small capacity of 58.1 mAh g^−1^ in BE at 20 A g^−1^, suggesting inferior rate performance especially at large current densities.

The long‐term cycling performance was further studied. The full cells using DE deliver a high capacity retention of 82 % after 800 cycles at 1 A g^−1^ and 83 % after 1,000 cycles at 10 A g^−1^ (Figure 5d and Figure S14). On the contrary, only 43 % of the initial capacity is retained when using BE at 1 A g^−1^ and a worse capacity retention of 32 % is delivered after 1,000 cycles at 10 A g^−1^. Furthermore, the Coulombic efficiency fluctuates drastically after long‐term cycling in BE at 1 A g^−1^, which is particularly obvious at large current density of 10 A g^−1^, indicating severe side reactions (Figure 5d and Figure S14). Therefore, a much better rate performance and cycling stability can be achieved in DE. In addition to excellent rate performance and cycling stability, the Zn||NH_4_V_4_O_10_ full cell in DE still exhibits a high capacity of 346.7 mAh g^−1^ at −15 °C and possesses an extraordinary capacity retention of 88 % after 640 cycles at low temperature (Figure 5e). Zinc cation coupled with chaotropic anion, CF_3_SO_3_^−^, have strong ability to break H‐bonds between water molecules, which leads to weaker H‐bond intensity and lower freezing point (Figure S15a). The lower freezing point correspondingly endows DE with effective ion migration ability for Zn plating/stripping and ions intercalation/deintercalation processes at low temperature (Figure S15b–c and Figure 5e).

**Figure 6:**
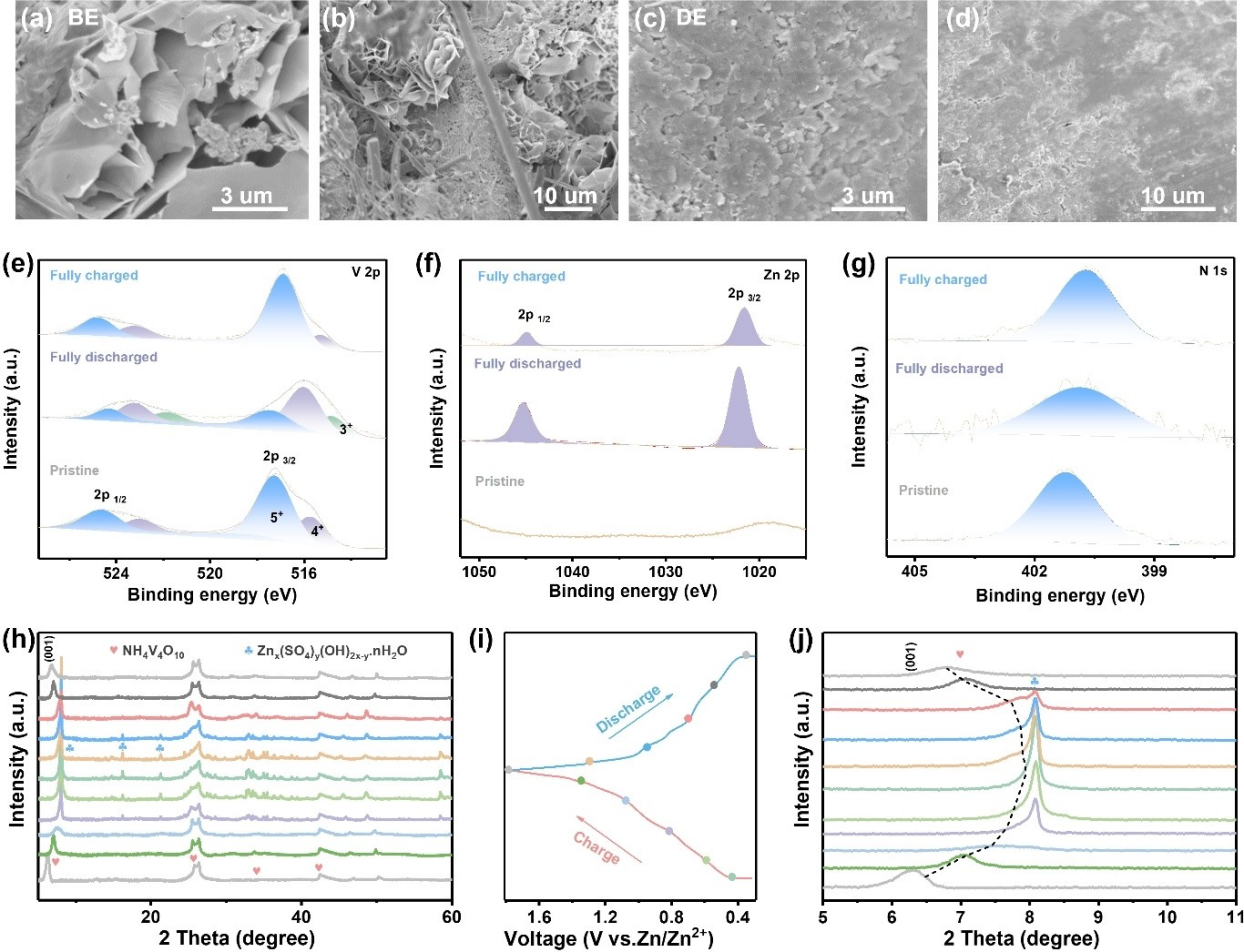
Charge storage mechanism characterizations. a–b SEM images of cycled Zn anode in BE at high and low magnifications, respectively. c–d SEM images of cycled Zn anode in DE at low and high magnifications, respectively. e–g Ex situ XPS spectra of V 2p, Zn 2p, and N 1s of NH_4_V_4_O_10_ cathodes in DE, respectively. h, j Ex situ XRD patterns of NH_4_V_4_O_10_ cathodes in DE at different charge/discharge states. i Corresponding GCD curves of (h) at 0.2 A g^−1^.

Multiple ex situ and in situ characterizations were performed to investigate the mechanism behind the electrochemical performance. As for the battery in DE, a corroded and rough Zn anode surface with obvious dendrites is observed after long‐term cycling (Figure 6a–b). The dendrite growth and Zn corrosion will perturb the reversibility of overall electrochemical reactions and therefore accelerate cell degradation.^[53]^ This also explains the Coulombic efficiency fluctuates and the capacity drastically fades in BE. By taking the advantage of the polymer‐inorganic SEI in DE, the Zn anode still exhibits a uniform and flat surface after long‐term cycling, suggesting greatly inhibited side reactions at the anode/electrolyte interface, which is beneficial for the overall battery performance (Figure 6c–d). Furthermore, XPS spectra was carried out to investigate the cathode side in DE. As shown in Figure 6f, no signal belonging to Zn^2+^ was detected in the pristine NH_4_V_4_O_10_ electrode. A substantive Zn 2p peak was observed in the fully discharged state, suggesting the successful insertion of Zn^2+^. Instead, a pair of imperceptible peaks related to Zn 2p signals were found in the fully charged state, which demonstrates the extraction of Zn^2+^.^[54]^ In the V 2p region, the peaks assigned to V^3+^ species increase, accompanied by a raise in V^4+^ and a drop in V^5+^ (Figure 6e). Subsequently, hybrid V species recovered to their original states in the fully charged state, suggesting that highly reversible redox reactions of V species occur during the intercalation/deintercalation of Zn^2+^.^[55]^ Moreover, the peaks related to NH_4_^+^ always exist in whole discharge/charge processes, indicating stable structural support within the VO_x_ polyhedral network (Figure 6g).^[56]^

After clarifying the variation of chemical states, ex situ XRD was used to analysis the crystal phase evolution of NH_4_V_4_O_10_ cathodes under different discharge/charge states. Figure 6h shows the ex situ characterized XRD patterns of NH_4_V_4_O_10_ cathodes in DE at different discharge/charge states according to the GCD curves (Figure 6i). The peak located at 6.4° representing the (001) crystal plane of NH_4_V_4_O_10_ shifts towards high 2θ values during the discharge process, and then recovers to its original state during the charge process, which indicates reversible cation intercalation/deintercalation and phase changes.^[57]^ Additionally, new peaks emerge at 8.1, 16.2, and 21.2 during discharge to low states, which can be indexed to Zn_x_(SO_4_)_y_(OH)_2x‐y_.nH_2_O, suggesting that OH^−^ ions react with the electrolyte and protons intercalation (Figure 6h and Figure 6j).^[58]^ It should be noted that, no peak belongs to Zn_x_otf_y_(OH)_2x‐y_.nH_2_O was detected in whole charge/discharge processes, further proving the preferential formation of Zn_x_(SO_4_)_y_(OH)_2x‐y_.nH_2_O in DE. To further gain insight into the cathode structure evolution, in situ Raman spectra were performed (Figure S16). The Raman peaks at 310–360 cm^−1^ and 574 cm^−1^ are related to the bending mode of the V−O−V bonds. The peaks at 760 cm^−1^ and 1031 cm^−1^ are derived from the stretching mode of the V=O bond.^[59]^ These peaks gradually weakened during the discharge process and then strengthened during the charge process, which is related to the Zn^2+^ intercalation/deintercalation.^[60]^ Hence, the reversible phase changes and Zn^2+^/H^+^ intercalation/deintercalation mechanism further validate a robust cycling performance of NH_4_V_4_O_10_ cathodes in the DE.

## Conclusion

In summary, a robust polymer‐inorganic hybrid SEI on Zn anode is constructed through controllable polymerization and coprecipitation mechanism. For the first time, the underlying SEI composition‐property‐performance relationships are systematically investigated and correlated. Compared with polymer SEI and inorganic SEI, only polymer‐inorganic SEI can combine the high modulus of the inorganic component with high toughness of the polymer ingredient, enabling the capacity of suppressing dendrites growth while accommodating the volumetric change. Therefore, Zn anode with this polymer‐inorganic SEI is able to realize high reversibility with an average coulombic efficiency of 99 % and sustain long‐term cycling over 4600 h. The long‐term cycling stability can still be maintained even under very harsh conditions of 30 mA cm^−2^ with an ultrahigh areal capacity of 30 mAh cm^−2^. The well‐designed polymer‐inorganic SEI further enables Zn||NH_4_V_4_O_10_ full cells with high capacity, excellent rate performance, and long‐term cycling stability. This work not only provides a novel strategy for building reliable hybrid SEIs on the Zn anode, but also discloses the SEI composition‐property‐performance relationships, which will aid the understanding, development, and application of SEIs in RAZBs.

## Conflict of interests

The authors declare no conflict of interest.

1

## Supporting information

As a service to our authors and readers, this journal provides supporting information supplied by the authors. Such materials are peer reviewed and may be re‐organized for online delivery, but are not copy‐edited or typeset. Technical support issues arising from supporting information (other than missing files) should be addressed to the authors.

Supporting Information

## Data Availability

The data that support the findings of this study are available from the corresponding author upon reasonable request.
