# Supplementary material for: Rational Design of an In‐Situ Polymer‐Inorganic Hybrid Solid Electrolyte Interphase for Realising Stable Zn Metal Anode under Harsh Conditions
Source: Angew Chem Int Ed Engl. 2024 Apr 18;63(21):e202401987. doi: 10.1002/anie.202401987 (PMC11497294; doi:10.1002/anie.202401987)
Supplement: Supplementary file 1 — Supporting Information [file ANIE-63-e202401987-s001.pdf]

Angewandte Chemie
Eine Zeitschrift der Gesellschaft Deutscher Chemiker

Supporting Information

Rational Design of an *In-Situ* Polymer-Inorganic Hybrid Solid Electrolyte Interphase for Realising Stable Zn Metal Anode under Harsh Conditions

R. Chen, W. Zhang, C. Guan, Y. Zhou, I. Gilmore, H. Tang, Z. Zhang, H. Dong, Y. Dai, Z. Du, X. Gao, W. Zong, Y. Xu, P. Jiang, J. Liu, F. Zhao, J. Li, X. Wang, G. He**

Supporting Information

Rational Design of An *In-Situ* Polymer-Inorganic Hybrid Solid Electrolyte Interphase for Realising Stable Zn Metal Anode under Harsh Conditions

Ruwei Chen,[a,b,#] Wei Zhang,[a,#] Chaohong Guan,[d,#] Yundong Zhou,[c] Ian Gilmore,[c] Hao Tang,[b] Zhenyu Zhang,[e] Haobo Dong,[a] Yuhang Dai,[a] Zijuan Du,[a] Xuan Gao,[a] Wei Zong,[a] Yewei Xu,[a] Peie Jiang,[a] Jiyang Liu,[a] Fangjia Zhao,[a] Jianwei Li,[a] Xiaohui Wang,[b,*] and Guanjie He[a,*]

[a] Dr. R. W. Chen, W. Zhang, Dr. H. B. Dong, Dr. Y. H. Dai, Z. J. Du, X. Gao, Dr. W. Zong, Dr. Y. W. Xu, P. E. Jiang, J. Y. Liu, F. J. Zhao, Dr. J. W. Li, Prof. G. J. He
Department of Chemistry
University College London
London WC1E 7JE, UK
E-mail: g.he@ucl.ac.uk

[b] Dr. R. W. Chen, H. Tang, Prof. X. H. Wang
State Key Laboratory of Pulp and Paper Engineering
South China University of Technology
Guangzhou 510641, China
E-mail: fewangxh@scut.edu.cn

[c] Dr. Y. D. Zhou, Prof. I. Gilmore
National Physical Laboratory
Hampton Road, Teddington TW11 0LW, UK

[d] C. H. Guan
University of Michigan-Shanghai Jiao Tong University Joint Institute
Shanghai Jiao Tong University
Shanghai 200240, China

[e] Dr. Z. Y. Zhang
Electrochemical Innovation Lab, Department of Chemical Engineering
University College London
London WC1E 7JE, UK

[#] These authors contributed equally to this work.

Experimental section

Electrolyte preparation: 2M $Zn(CF_3SO_3)_2$ electrolyte was prepared by dissolving 0.2 mol $Zn(CF_3SO_3)_2$ (Sigma-Aldrich) into 100 mL deionized water, which is denoted as BE. The polymer SEI forming electrolyte was then prepared by mixing proper amount of acrylamide with the as-prepared 2M $Zn(CF_3SO_3)_2$ solution to control the concentration of acrylamide additive (Sigma-Aldrich) as 0.2 M. The inorganic SEI forming electrolyte was then prepared by mixing proper amount of $ZnSO_4$ (VWR chemicals) with the as-prepared 2M $Zn(CF_3SO_3)_2$ solution to control the concentration of $ZnSO_4$ additive as 0.2 M. The polymer-inorganic SEI forming electrolyte was prepared by mixing proper amounts of acrylamide and $ZnSO_4$ with the as-prepared 2M $Zn(CF_3SO_3)_2$ solution to control the concentrations of acrylamide and $ZnSO_4$ additive as 0.2 M, which is denoted as DE.

Electrode preparation: $NH_4V_4O_{10}$ cathode material was prepared by a single-step hydrothermal reaction according to our previous work.[1] In a typical synthesis process, 5 mmol of ammonium metavanadate was dissolved into 30 ml of deionized water with 10 min stirring under ambient environment. After that, a mixture of 6 mmol of oxalic acid and 1 mmol of ammonium fluoride were added. Then, the mixed solution was transferred into 50 ml Teflon-lined autoclaves and heat up to 180 °C for 6 hours. The as-obtained bronze-color precipitates were washed by deionized water for several times. Finally, the washed sample was freeze-dried for 2 days. The cathode electrodes were fabricated by mixing polyvinylidene fluoride (Sigma-Aldrich), Super-P (Sigma-Aldrich) and as-prepared materials with a ratio of 1:2:7 using N-methyl-2-pyrrolidone as the solvent. The mixture slurry was printed on a hydrophilic carbon paper and then transferred to a vacuum oven to dry under 80 °C for 10 hours. The mass loading of the cathode was about 1.2~1.5 $mg\cdot cm^{-2}$.

Materials characterizations: SEM was conducted on JEOL-JSM-6700F. The XRD patterns were performed on a PANalytical Empyrean device with Cu K_α radiation. A LabRAM HR Evolution instrument (laser wavelength: 532 nm) was carried out to collect the Raman spectra. FTIR-spectra were conducted on an ATR-FTIR (BRUKER,

platinum-ATR). X-ray photoelectron spectroscopy were collected by a Thermo scientific K-alpha photoelectron spectrometer.

AFM experiments was conducted according to previous work.[2] Specifically, a Bruker dimension Icon with ScanAsyst device was employed to conduct AFM experiments. PeakForce tapping mode was adopted in all of the AFM imaging with an RTESPA-300 silicon probe with reflective Al coating (Bruker Corp., $k = 40$ N m^{-1}, $f_0 =$ 300 kHz). Before every experiment, the probe was calibrated by using a standard highly oriented pyrolytic graphite (modulus = 18 GPa) sample for a precise measurement of the mechanical properties. Nanoindentation mechanical measurements were conducted at the same time as the morphology was mapped. By recording the load and displacement of the specialized tips and cantilevers when being pressed into the surface a load−displacement curve could be generated, which was further used to calculate the hardness and elastic modulus of the cycled Zn anodes. All of the results obtained by AFM were analyzed by Nanoscope Analysis software. To obtain the Young's Modulus, the retract curve is fit using the Derjaguin-Muller-Toporov (DMT) model.

$$F - F_{adh} = \frac{4}{3}E^{*}\sqrt{R(d-d_0)^3}$$

Where $F\text{-}F_{adh}$ is the force on the cantilever relative to the adhesion force, R is the tip end radius, and $d\text{-}d_0$ is the deformation of the sample. The result of the fit is the reduced modulus E^{*}. The software can calculate the Young' Modulus of the sample E_s by the equation:

$$E^{*} = \left[\frac{1-v_s^2}{E_s} + \frac{1-v_{tip}^2}{E_{tip}}\right]^{-1}$$

Where E_{tip} is the Young's modulus of silicon tip, v_s and v_{tip} are Poisson's ratio of graphite and silicon probe, respectively.

ToF-SIMS 3D mapping was carried out using a TOF-SIMS V instrument (IONTOF GmbH, Germany). The ToF-SIMS data were acquired in negative ion polarity mode by raster scanning a 30 keV Bi_3^+ primary ion beam over an analytical area of 200 μm × 200 μm with a beam current of 0.39 pA. A 20 keV gas cluster ion beam (GCIB) with a current of 17 nA was used to sputter remove material with a sputter

area of 300 μm × 300 μm centred with the analytical area in non-interlaced model. A low-energy (20 eV) electron flood gun was employed to neutralise charge build up. The mass spectrum was calibrated with C^-, CH^-, C_2^-.

Electrochemical tests: The electrochemical performances of Zn//Zn symmetric cells, Zn//Cu asymmetric cells, Zn//$NH_4V_4O_{10}$ full cells were tested using CR 2025 coin cells. The evaluation of cyclic voltammetry, chronoamperometry measurement, and electrochemical impedance spectroscopy (EIS) tests were achieved by a VMP3 Biologic potentiostat. The evaluation of galvanostatic charge-discharge was tested by a Neware battery testing system. All Zn foil (99.9%) anodes were utilized after polished by 1000 grit sandpaper. Glass-fiber (GF/A, Whatman) was chosen as the separator. To test the activation energy, electrochemical impedance spectroscopy was conducted at different temperatures ranging from 303.15 to 353.15 K. Then, the activation energy was calculated by Arrhenius equation:

$$-lnR_{ct} = lnA - \frac{E_a}{RT}$$

where, R_{ct} is the charge-transfer resistance, A is the frequency factor, and E_a is the activation energy related to the zinc-ion transport and the desolvation process.

Zn^{2+} transference number ($t_{Zn^{2+}}$) was measured by the potentiostatic polariztion method based on Zn//Zn symmetric cell and calculated with Evans equation listed below:

$$t_{Zn^{2+}} = \frac{I_S(\Delta V - I_0R_0)}{I_0(\Delta V - I_sR_s)}$$

where I_0 and I_s represent the currents at the initial and steady states, respectively. R_0 and R_s represent the interfacial resistances before and after polarization, respectively. These results were obtained by performing EIS test before and after chronoamperometry measurements.

Computational Details

All quantum chemical calculations were performed by applying the density functional theory (DFT) method with the B3LYP level and 6-31+G(d,p) basis set using Gaussian package [3]. The structural optimization of the molecules or ions in this work was determined by minimizing the energy without imposing molecular symmetry

constraints. The transition state was determined by the QST2 method and was confirmed by the IRC calculations so that the reaction barriers can be obtained. In addition, the QUANTUM ESPRESSO was adopted to study the binding energies [4]. The Perdew-Burke-Ernzerhof (PBE) functionals based on the generalized-gradient approximation (GGA) was used to describe the exchange-correlation energy and the Van der Waals interactions were considered by the DFT-D2 correction introduced in Moellmann and Grimme [5]. The energy cutoff was set to 40 Ry, and the electron convergence threshold was taken to by 10-6 Ry, the 2 × 2 ×1 Monkhorst-Pack k-point grid was sampled for the optimizing models. The binding energy can be defined as:

$$E_b = E_{total} - E_{PAM} - E_{molecule}$$

Where E_{total} is the total energy of the molecule adsorption on the PAM, E_{PAM} and $E_{molecule}$ are the energy of PAM and adsorbed molecule, respectively. Besides, the CINEB method [6] was used to calculate the energy barriers of Zn in the $Zn_4(OH)_6SO_4 \cdot 5H_2O$.

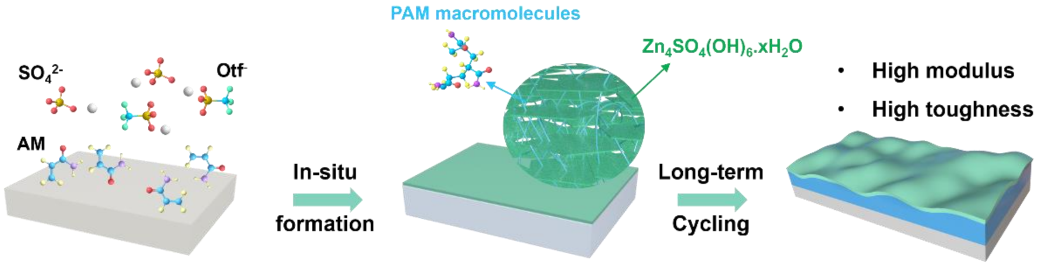

Figure S1. Schematic illustration of the *in-situ* formed polymer-inorganic SEI.

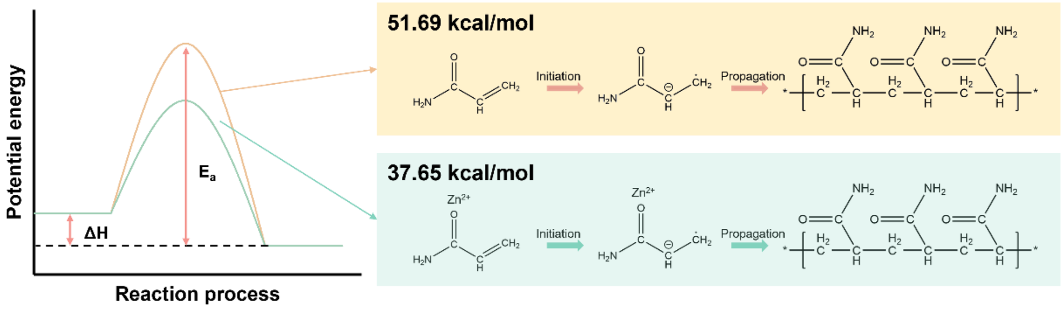

Figure S2. Schematic diagram of activation energy barriers and corresponding reaction free energies of AM polymerization with or without Zn^{2+}.

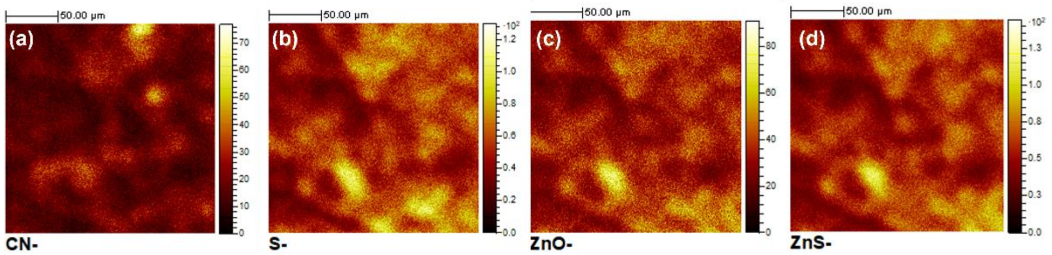

Figure S3. Chemical ion images of the CN^-, S^-, ZnO^-, and ZnS^- species for the polymer-inorganic SEI.

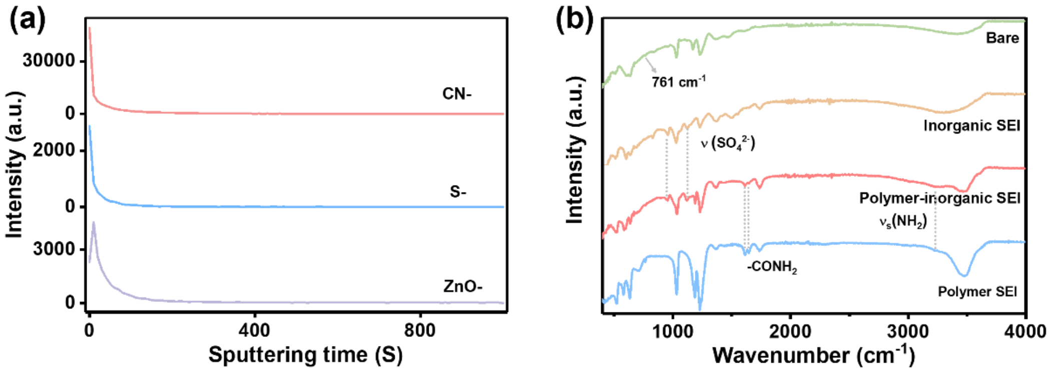

Figure S4. (a) Intensity profiles of CN^-, S^-, and ZnO^- for bare Zn. (b) FTIR spectra of Zn anodes with different SEIs.

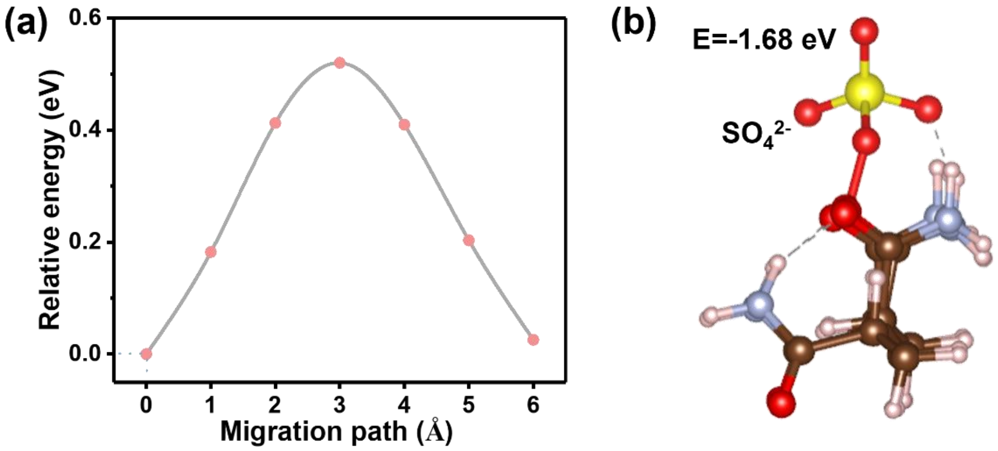

Figure S5. Working mechanism of the polymer-inorganic SEI layer. (a) Corresponding diffusion energy barriers of Zn^{2+} in ZHS. (b) DFT calculation of the interactions of foreign SO_4^{2-} on PAM polymer.

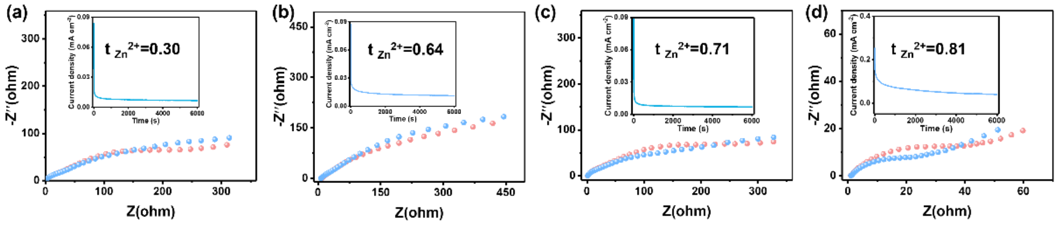

Figure S6. EIS spectra of different symmetric cells before and after polarization. (a) Bare Zn. (b) Polymer SEI. (c) Inorganic SEI. (d) Polymer-inorganic SEI.

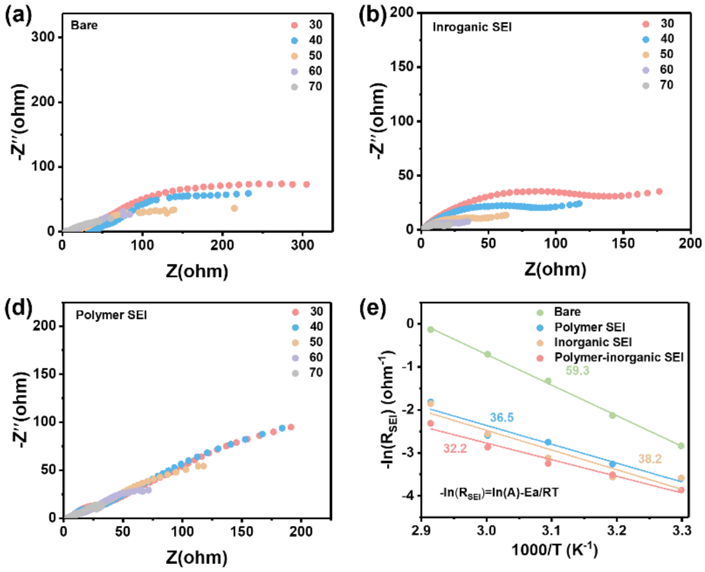

Figure S7. EIS spectra of different symmetric cells at various temperatures. (a) Bare Zn. (b) Inorganic SEI. (c) Polymer-inorganic SEI. (d) Polymer SEI. (e) Arrhenius curves of different symmetric cells: activation energy.

Table S1. Electrochemical performance of Zn//Zn symmetric cells with polymer-inorganic SEI.

Current density (mA cm^{-2})	Areal capacity (mAh cm^{-2})	Lifespan (h)	Cumulative capacity (mAh cm^{-2})	J*C (mA cm^{-2}*mAh cm^{-2})
1	1	4605	2302.5	1
3	3	1560	2340	9
5	5	1325	3312.5	25
10	10	870	4350	100
20	20	341	3410	400
30	30	200	3000	900

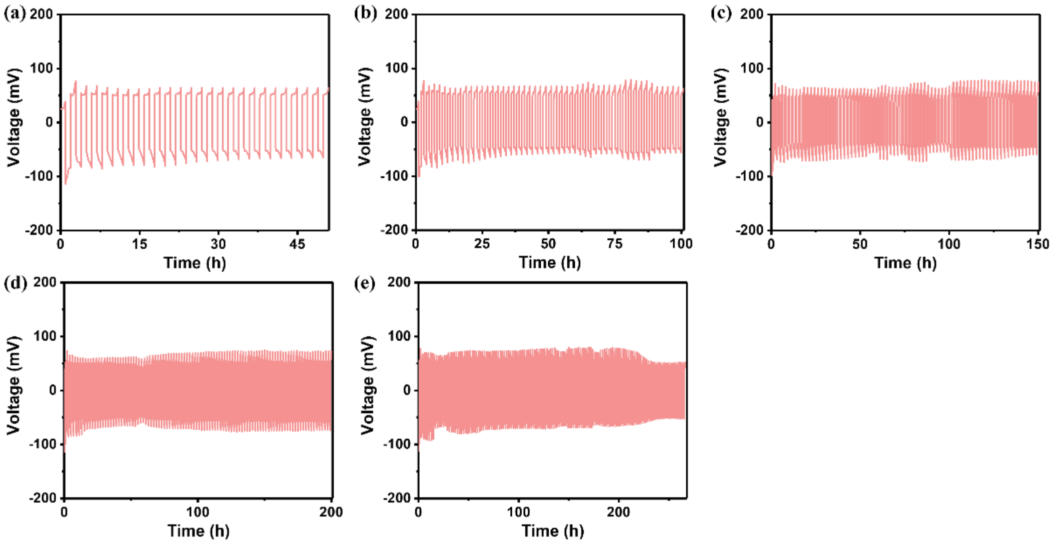

Figure S8. Voltage profiles of Zn||Zn symmetric cells with polymer-inorganic SEI cycled at a current density of 10 mA cm^{-2} and an areal capacity of 10 mAh cm^{-2} for different time: (a) 50 h, (b) 100 h, (c) 150 h, (d) 200 h, (e) 260 h.

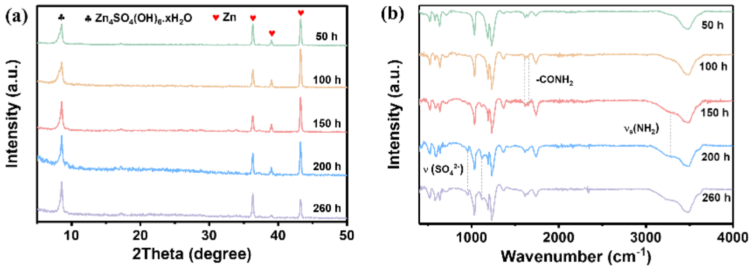

Figure S9. (a) XRD patterns of Zn anodes with polymer-inorganic SEI after cycling for different time. (b) FTIR spectra of Zn anodes with polymer-inorganic SEI after cycling for different time.

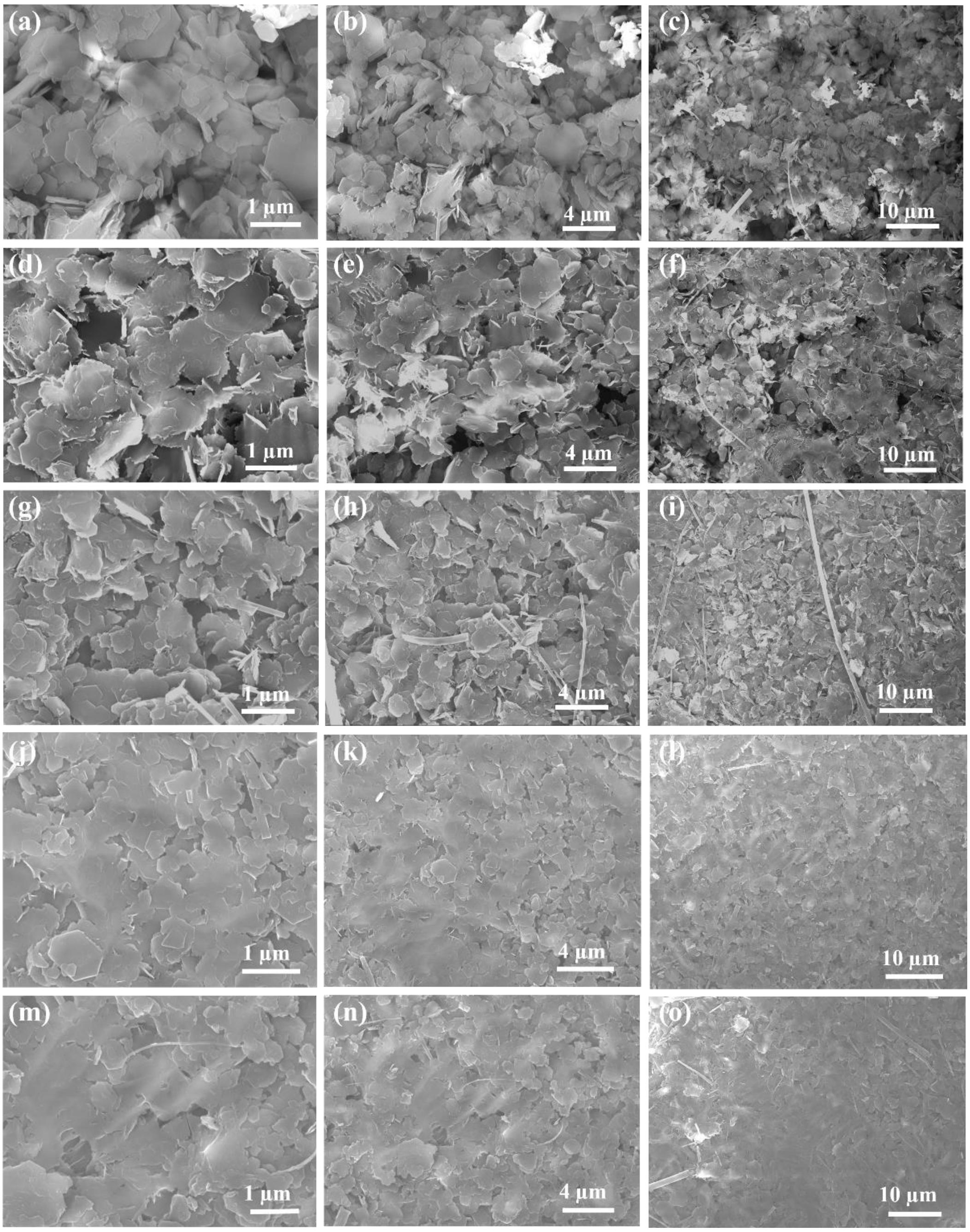

Figure S10. SEM images of Zn anodes with polymer-inorganic SEI after cycling for different time at a current density of 10 mA cm^{-2} and an areal capacity of 10 mAh cm^{-2}. (a-c) SEM images of Zn anodes after cycling for 50 h. (d-f) SEM images of Zn anodes after cycling for 100 h. (g-i) SEM images of Zn anodes after cycling for 150 h. (j-l) SEM images of Zn anodes after cycling for 200 h. (m-o) SEM images of Zn anodes after cycling for 250 h.

Figure S8 shows the voltage profiles of Zn||Zn symmetric cells with polymer-inorganic SEI cycled at a current density of 10 mA cm^{-2} and an areal capacity of 10 mAh cm^{-2} for different time. The voltage profiles of symmetric cells show a trend of first increasing and then decreasing as the cycle time increases, which is consistent with the evolution trend in Figure 3c. After cycling for different time, the Zn||Zn symmetric cells were disassembled for further characterization. As shown in Figure S9a, except for peaks index to zinc, all other peaks belong to inorganic ZHS. No peaks belonging to other substances were found as the cycle time increased. Moreover, the characteristic peaks indexed to SO_4^{2-}, $-NH_2$, and $-CONH_2$ are observed for all samples in FTIR spectra, which is consistent with our previous data and confirms that the SEI composition did not change during the cycling process (Figure S9b). SEI-involved electrode/electrolyte interface plays a critical role in Zn plating/stripping behaviors. Herein, SEM images associate with electrochemical voltage profiles are provided. Figure S10 shows the corresponding SEM images of Zn anodes with polymer-inorganic SEI after cycling for different time. By taking advantage of our well-designed electrolyte, a polymer-inorganic SEI was in-situ constructed on Zn anode, where the constructed SEI gradually evolves with increasing cycle time. The trend of SEI evolution is consistent with the changes in the corresponding voltage profiles. As a result, a more uniform and compact SEI layer was generated on Zn anode with increasing cycle time at initial period, which is more conducive to the stability of electrode/electrolyte interface and better Zn plating/stripping behaviors. In addition, the characteristic peaks belonging to inorganic ZHS and organic PAM slightly strengthen with the increase of cycle time, which further confirms our above conclusion (Figure S9). On the other hand, the increasing Coulombic efficiency of Zn||Cu cell with polymer-inorganic SEI also indicates the evolution of a more uniform, stable, and compact SEI (Figure 3a).

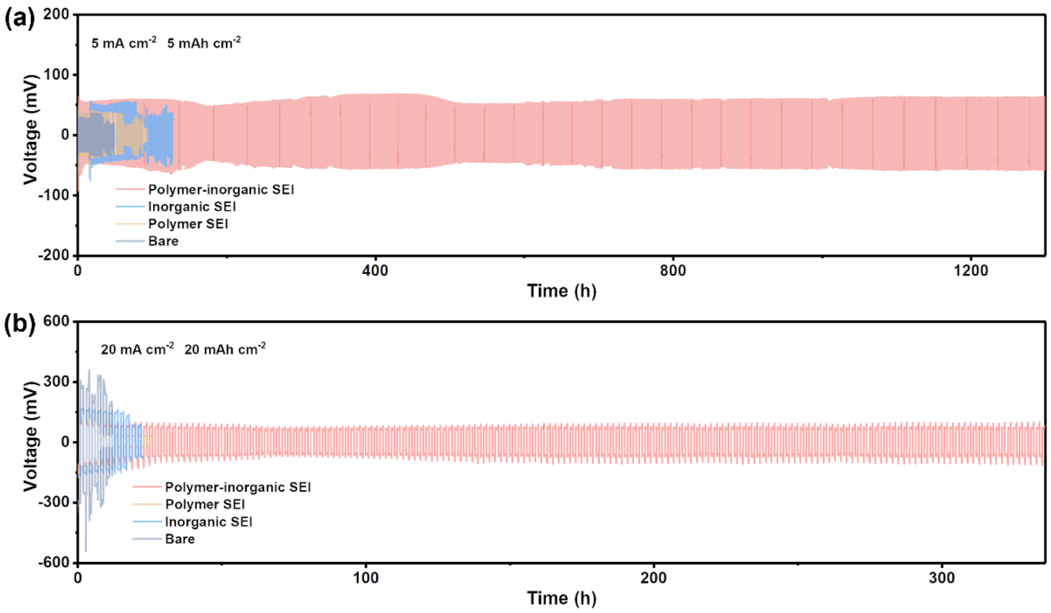

Figure S11. Long-term cycling performance of Zn//Zn symmetric cells at different current densities and areal capacities: (a) 5 mA cm^{-2}-5 mAh cm^{-2}, (b) 20 mA cm^{-2}-20 mAh cm^{-2}.

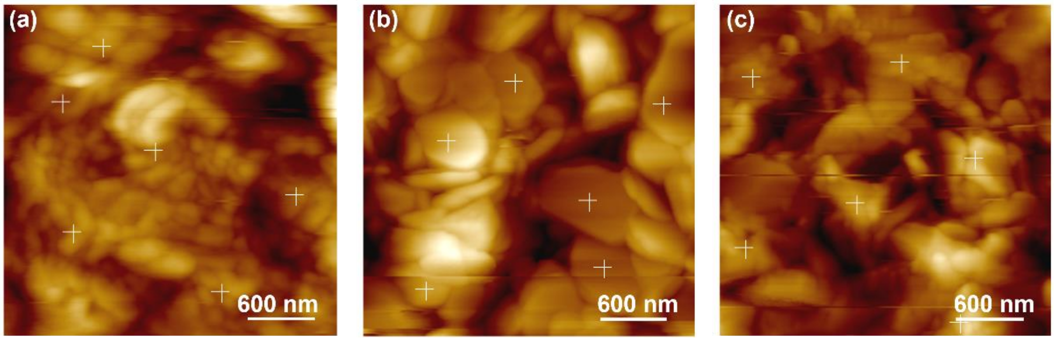

Figure S12. Six spots of the force-distance curves. (a) Polymer SEI. (b) Inorganic SEI. (c) Polymer-inorganic SEI.

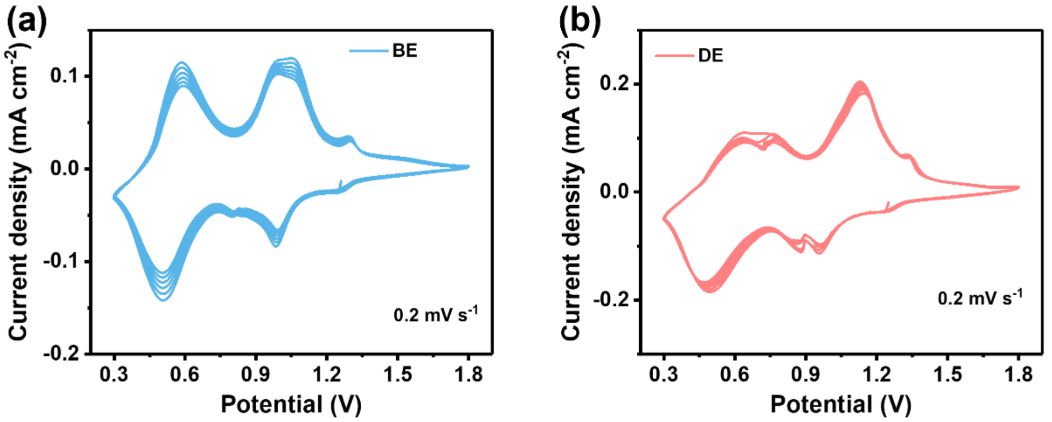

Figure S13. CV curves of Zn||$NH_4V_4O_{10}$ batteries at 0.2 mV s^{-1} in (a) BE and (b) DE.

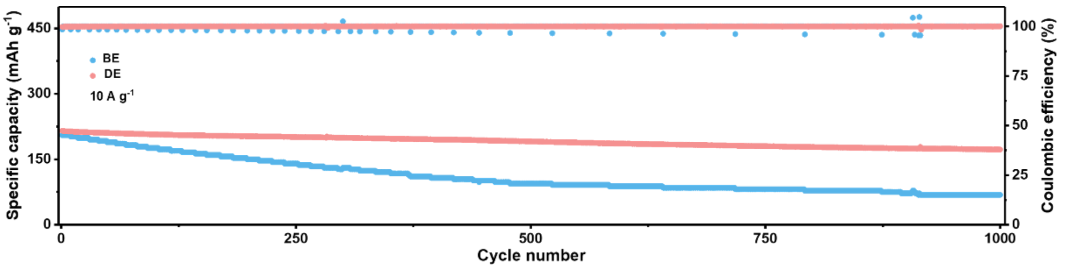

Figure S14. Comparison of the cycling performance at 10 A g^{-1}.

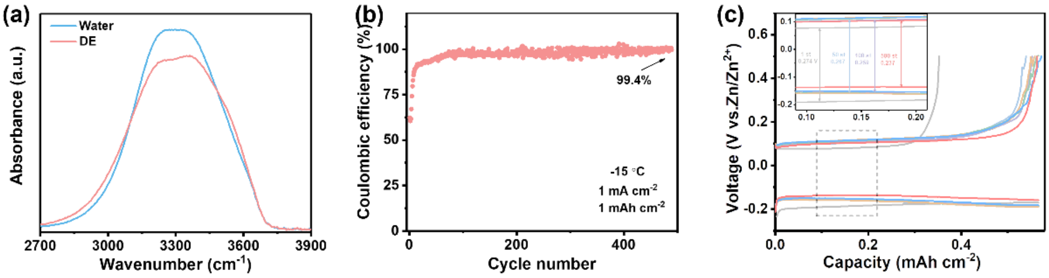

Figure S15. (a) FTIR spectra of water and DE. (b) Coulombic efficiency of Zn//Cu asymmetric cell in DE at low temperature. (c) Corresponding voltage profiles of Zn//Cu asymmetric cell in DE at low temperature.

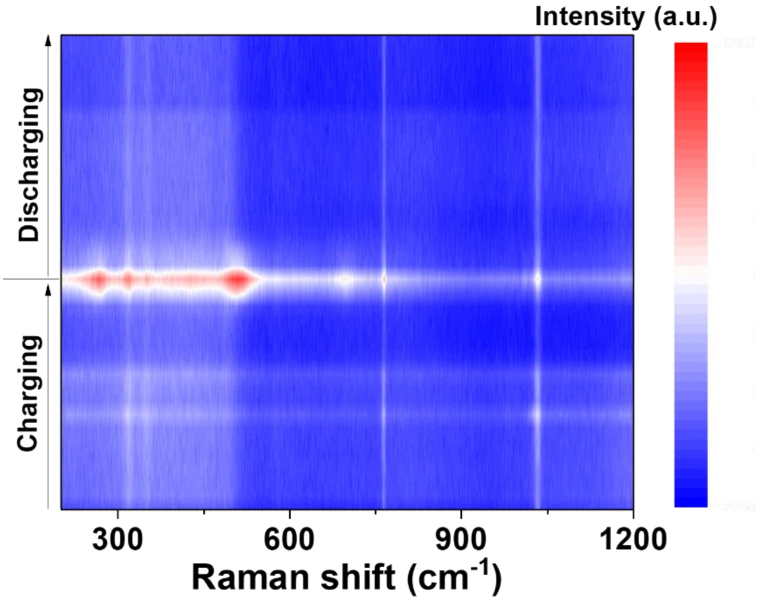

Figure S16. In-situ Raman spectra of $NH_4V_4O_{10}$ cathode in DE.

References

[1] J. Li, N. Luo, F. Wan, S. Zhao, Z. Li, W. Li, J. Guo, P. R. Shearing, D. J. L. Brett,C. J. Carmalt, G. Chai, G. He, I. P. Parkin, *Nanoscale* **2020**, 12, 20638.

[2] Z. Zhang, K. Smith, R. Jervis, P. R. Shearing, T. S. Miller, D. J. L. Brett, *ACS Appl Mater Interfaces* **2020**, 12, 35132.

[3] M. J. Frisch, G. W. Trucks, H. B. Schlegel, G. E. Scuseria, M. A. Robb, J. R. Cheeseman, G. Scalmani, V. Barone, G. A. Petersson, H. Nakatsuji et al., Gaussian 16, Gaussian, Inc., Wallingford, CT, 2016.

[4] Giannozzi P, Baroni S, Bonini N, et al. Quantum Espresso: a modular and open-source software project for quantum simulations of materials. J Phys Condens Matter 2009; 21:395502.

[5] Grimme S. Semiempirical GGA-type density functional constructed with a long-range dispersion correction. J. Comput. Chem. 2006; 27:1787–1799.

[6] Henkelman, G.; Jónsson, H. Improved tangent estimate in the nudged elastic band method for finding minimum energy paths and saddle points. J. Chem. Phys. 2000, 113, 9978−9985.
